# Zinc biofortification through seed nutri-priming using alternative zinc sources and concentration levels in pea and sunflower microgreens

**DOI:** 10.3389/fpls.2023.1177844

**Published:** 2023-04-17

**Authors:** Pradip Poudel, Francesco Di Gioia, Joshua D. Lambert, Erin L. Connolly

**Affiliations:** ^1^ Department of Plant Science, The Pennsylvania State University, University Park, PA, United States; ^2^ Department of Food Science, The Pennsylvania State University, University Park, PA, United States

**Keywords:** agronomic biofortification, antinutrients, nutrient priming, micronutrients, phytic acid, phytochemical analysis, seed soaking

## Abstract

Micronutrient deficiencies caused by malnutrition and hidden hunger are a growing concern worldwide, exacerbated by climate change, COVID-19, and conflicts. A potentially sustainable way to mitigate such challenges is the production of nutrient-dense crops through agronomic biofortification techniques. Among several potential target crops, microgreens are considered suitable for mineral biofortification because of their short growth cycle, high content of nutrients, and low level of anti-nutritional factors. A study was conducted to evaluate the potential of zinc (Zn) biofortification of pea and sunflower microgreens *via* seed nutri-priming, examining the effect of different Zn sources (Zn sulfate, Zn-EDTA, and Zn oxide nanoparticles) and concentrations (0, 25, 50, 100, and 200 ppm) on microgreen yield components; mineral content; phytochemical constituents such as total chlorophyll, carotenoids, flavonoids, anthocyanin, and total phenolic compounds; antioxidant activity; and antinutrient factors like phytic acid. Treatments were arranged in a completely randomized factorial block design with three replications. Seed soaked in a 200 ppm ZnSO_4_ solution resulted in higher Zn accumulation in both peas (126.1%) and sunflower microgreens (229.8%). However, an antagonistic effect on the accumulation of other micronutrients (Fe, Mn, and Cu) was seen only in pea microgreens. Even at high concentrations, seed soaking in Zn-EDTA did not effectively accumulate Zn in both microgreens’ species. ZnO increased the chlorophyll, total phenols, and antioxidant activities compared to Zn-EDTA. Seed soaking in ZnSO_4_ and ZnO solutions at higher concentrations resulted in a lower phytic acid/Zn molar ratio, suggesting the higher bioaccessibility of the biofortified Zn in both pea and sunflower microgreens. These results suggest that seed nutrient priming is feasible for enriching pea and sunflower microgreens with Zn. The most effective Zn source was ZnSO_4_, followed by ZnO. The optimal concentration of Zn fertilizer solution should be selected based on fertilizer source, target species, and desired Zn-enrichment level.

## 1 Introduction

Zinc (Zn) is a micromineral essential for human health and is involved in various physiological processes, including gene regulation and metabolic pathways (Chasapis et al., 2020; Wessels et al., 2022). Zinc deficiency could lead to physiological dysfunctions, disease, and even death, especially in the case of children and elderly people, as they are most susceptible to the “hidden hunger” problem due to inadequate intake and absorption. Approximately 17.1% of the global population is estimated to be at risk of inadequate Zn intake (Wessells and Brown, 2012), and factors such as the growing world population, climate change, infectious disease pandemics like COVID-19, and conflicts could further exacerbate the issue of Zn deficiency (Beach et al., 2019; Wessels et al., 2022). Zinc deficiency is a malnutrition issue most prevalent in low- and middle-income countries due to limited access to Zn-rich foods, a less diverse diet, and dependency on a cereal-based diet with low Zn bioavailability due to phytate binding (Gupta et al., 2020). Moreover, low Zn soil levels have also been linked to the prevalence of the deficiency, mainly in developing countries (De Groote et al., 2021). Zinc deficiency is also a problem in developed countries like the USA. For example, 15% of the US adult population consume lower Zn than the estimated average requirement (EAR) (Reider et al., 2020). The leading cause of Zn deficiency in developed countries is the dependency on food characterized by high-calorie density but a lower bioavailable Zn content (Kruger et al., 2014).

Different approaches have been proposed to mitigate hidden hunger problems like Zn deficiency, including increasing the proportion and diversity of vegetables in the diet, fortification of foods, nutrient supplementation, and crop biofortification. Among these mitigation strategies, diversification of diet and biofortification with Zn are considered the most sustainable approach to address Zn deficiency in susceptible populations. Crops can be biofortified through different approaches, including genetic engineering, conventional breeding, and agronomic crop management (Di Gioia et al., 2019; Marques et al., 2021). Agronomic biofortification has advantages over other approaches in that it can be implemented in a wide range of already adopted crops and cultivars. Therefore, it could help address malnutrition issues under different circumstances relatively quickly (Di Gioia et al., 2019; Buturi et al., 2021; Marques et al., 2021).

One approach to biofortify vegetables *via* agronomic biofortification in vegetable crops is through Zn-enriched nutrient solution application to seeds prior to planting. There are various sources of Zn on the market, and they come in multiple forms, including inorganic salts, nanoparticles, and chelated forms. Zinc sulfate (ZnSO_4_) is the most popular salt because it is relatively inexpensive, highly soluble, and widely available on the market (Veena and Puthur, 2022). Chelated synthetic fertilizers, such as Zn-EDTA, are also popular sources. Owing to the gradual release in the soil/nutrient matrix, Zn-EDTA increases the efficiency of plant absorption, particularly under alkaline conditions, and inhibits the development of the insoluble complex (Zhao et al., 2018). Chelated fertilizers generally are more expensive than inorganic alternatives.

Additionally, research has demonstrated that the rate at which plants absorb Zn varies depending on the type of Zn applied, although the results reported in the literature to date are inconsistent. For example, Montanha et al. (2020) reported a higher uptake of Zn in soybean plants from Zn sulfate than Zn-EDTA, while higher Zn uptake and concentration in the wheat plant was found by Zhao et al. (2018) with Zn-EDTA in soil. Zinc oxide (ZnO) nanoparticles are another alternative source of Zn fertilizer that is now under evaluation. Because of their unique qualities, such as very small size (1–100 nm), high surface/volume ratios, increased stability, and reactivity, they may have higher absorption efficiency (De La Torre-Roche et al., 2020; Veena and Puthur, 2022). Nanoparticles may not only increase the concentration of specific minerals but also affect the plant phytochemical profiles and increase crop nutritional value (García-López et al., 2019; Quiterio-Gutiérrez et al., 2019). For example, cucumber leaves and roots exhibited enhanced nutrient uptake (N, P, K, and Zn), chlorophyll, carotenoids, total phenols, flavonoids, and antioxidant activity following foliar treatment with ZnO nanoparticles three times per week for 2 weeks, beginning 34 days after transplanting (Ghani et al., 2022).

Among the alternative agronomic biofortification methods, seed nutri-priming is a simple, easy-to-use technique that involves soaking seeds in a solution containing macro- and/or micronutrients before sowing. Most nutri-priming research has been done on cereals and leguminous grain crops to increase germination, seed vigor, growth under stress conditions, and biofortification purposes (Farooq et al., 2019). The biofortification target, in this case, is the grain produced by plants derived from nutri-primed seeds. Seed nutri-priming has also been proposed for enhancing the bioaccessibility and bioavailability of minerals such as Fe and Zn in soybean sprouts (Zou et al., 2014). An interesting aspect to consider in this case is that during the nutrient priming process, soaking the seeds can reduce the concentration of antinutrient compounds, such as phytic acid, *via* leakage (West et al., 1994; Egli et al., 2002). Very little research is available on the biofortification of vegetables *via* nutrient priming (Zou et al., 2014; Przybysz et al., 2016; Bączek-Kwinta et al., 2020). Microgreens could be an excellent choice for nutrient biofortification by nutrient priming since they are consumed at an early seedling stage, and nutrients can rapidly translocate from seed to greens (Di Gioia et al., 2021). Furthermore, microgreens have high nutrient content and low antinutrient compounds like phytate (Kyriacou et al., 2016; Di Gioia et al., 2017; Di Gioia et al., 2021). We hypothesize that soaking seeds in a concentrated nutrient solution before seeding could enrich microgreens with specific minerals. However, research is needed to define the optimum nutrient concentration and fertilizer source for the biofortification of different microgreens species. Therefore, the present study aims to evaluate the potential of Zn biofortification in two commonly grown microgreen species using the seed nutri-priming technique with three sources of Zn and assess the impact on their yield and nutritional profile.

## 2 Materials and methods

### 2.1 Experiment site, experimental design, and treatments

The experiment was conducted, during the spring of 2021, in a glasshouse at the Pennsylvania State University Greenhouse Facilities in Central Pennsylvania at University Park, PA.

Two commonly consumed microgreen species, “Dwarf grey sugar” peas (*Pisum sativum* L.) and “Black oil” sunflower (*Helianthus annuus* L.), were selected for the study. Seeds were purchased from Johnny’s Selected Seeds (Winslow, ME, USA). The germination percentage of the purchased seeds was 96% and 90% for peas and sunflowers, respectively. Seeds were soaked in different nutrient solutions.

A completely randomized factorial block plus control design was used for the study with two levels of species, three levels of Zn sources, and four levels of the Zn concentration. Zinc nutrient sources used for the study were Zn sulfate (ZnSO_4_H_2_O, Alpha Chemicals, Cape Girardeau, MO, USA), Zn-EDTA (14% chelated Zn, Greenway Biotech, Santa Fe Springs, CA, USA), and Zn oxide nano-powder (ZnO, 99.9+%, 80–200 nm, US Research Nanoparticles, Inc, Houston, TX, USA). For each source of Zn, deionized (DI) water solutions with four levels of Zn concentration were tested (25, 50, 100, and 200 mg/l), while DI water was used as a control treatment. Altogether, there were 12 treatment combinations plus control for each species for a total of 26 treatments; each treatment was replicated three times. Seeds were soaked in the respective Zn water solution/emulsions for 12 h overnight before seeding. Nutrient solutions/emulsions were prepared to dissolve the Zn fertilizer in DI water and properly mix the solution. The saturated solution was aerated using hydroponic air pumps throughout the seed soaking period to provide oxygen to the seeds, continue mixing the nutrient solution, and avoid any precipitation.

### 2.2 Growing system, seed sowing, and harvest

Seeds were seeded in small growing trays (12 cm × 16 cm) filled with a commercial peat-perlite mix (Sunshine Mix 4, Sun Gro Horticulture, Agawam, MA, USA) at a seed density of 1 seed/cm^2^. There were three small trays per experimental unit with three replications for nine trays per treatment. After sowing, a weight was placed on the trays to press seeds into the growing media, and the growing trays were covered with a black plastic film to create a dark environment during germination. After the germination of the seeds, the weights and plastic covers were removed. Growing trays were misted with DI water every day until the cotyledon formation, and after that, growing media were watered from the bottom. Supplemental LED light (Illumitex ES24812 Eclipse Surexi Double Bar LED Grow Lights) was provided from 6:00 a.m. to 9:00 p.m. when solar radiation was below 1,000 mW. During the microgreen growing period, the average greenhouse temperature was set at 25.3°C, with minimum and maximum temperatures set at 23.8 and 26.6°C. Relative humidity ranged between 10% and 65%. Sunflower and pea microgreens were harvested 9 and 10 days after sowing, respectively. Total fresh weight and mean fresh shoot weight were measured at harvest, and subsamples were either oven-dried until constant weight at 65°C to measure dry weight and dry matter content and analyze minerals or stored at −80°C and freeze-dried for nutritional analysis.

### 2.3 Mineral analysis

Oven-dried ground samples were sent to the Penn State Agricultural Analytical Services Laboratory at University Park, PA, for mineral analysis. Samples were analyzed for total nitrogen using dry combustion with an Elementar Max Cube in CN mode (Elementar Americas Inc., Ronkonkoma, NY) as described in Vecchia et al. (2020) and macrominerals (P, K, Ca, Mg, S, and Na) and microminerals (Mn, Fe, Cu, B, and Zn) after acid digestion (Huang and Schulte, 1985) using an ICP-OES (Varian 730-ES, Agilent Technologies, Santa Clara, CA, USA).

### 2.4 Phytochemical analysis

#### 2.4.1 Total chlorophyll and carotenoids

Total chlorophyll and carotenoid content in the sample were analyzed using the method explained by Porra et al. (1989) with slight modifications (Lee et al., 2021b). A freeze-dried ground sample (0.015 g) was extracted with 1.5 ml of 80% acetone for 25 min in an ultrasonic processor and then with 100 vol. of 80% methanol for 25 min (Branson CPX2800H, Branson Ultrasonics, Brookfield, CT). The mixed sample was then centrifuged at 4,000 × *g* for 5 min. The absorbance of the supernatant at 663 nm (A663), 645 nm (A645), and 470 nm (A470) was recorded in a microplate reader (Synergy H1, BioTek, Winooski, VT).

#### 2.4.2 Flavonoids and anthocyanin

Flavonoid was analyzed using an AlCl_3_ colorimetric assay (Herald et al., 2012). For sample extraction, 0.04 g of the freeze-dried ground sample was mixed with 100 vol. of the 80% methanol in the falcon tube and placed in a sonicator (Branson CPX2800H, Branson Ultrasonics, Brookfield, CT) for 20 min after vortexing for 20 s. Samples were then extracted overnight at 4°C in the dark. The sample extract was centrifuged at 12,000 × *g* for 2 min before analyzing the flavonoid content. The following chemicals were added sequentially in microplate wells: 100 µl of distilled water, 10 µl of NaNO_2_ (0.73 M NaNO_2_), 25 µl of sample solution, and standard or methanol (for blank). After 5 min of waiting, 15 µl of AlCl_3_ (0.75 M AlCl_3_) was added, and the reaction was run for 6 min, followed by adding 50 µl of NaOH (1 M NaOH) and 50 µl of distilled water in sequence. The microplate was shaken for 2 min, and absorbance was read at 510 nm in a microplate reader (Synergy H1, BioTek, Winooski, VT). Catechin was used as a standard (10–640 µg/ml), and total flavonoid content was quantified based on catechin equivalent (CE) based on dry weight (mg CE/g DW).

Anthocyanin content was analyzed following the method described by Nakata and Ohme-Takagi (2014) with modification (Lee et al., 2021b). A freeze-dried ground sample (0.02 g) was mixed with the 25 vol. of extractant solvent (methanol:acetic acid, 45:5 v/v) and vortexed for 10 s. After vortexing, the sample was placed into an ultrasonic processor for 20 min. The supernatant was transferred to another centrifuge tube and centrifuged at 12,000 × *g* for 5 min. The supernatant (300 µl) or extraction solvent (for blank) was then transferred to the microplate well for reading absorbance at 530 nm and 657 nm in a microplate reader (Synergy H1, BioTek, Winooski, VT).

#### 2.4.3 Total phenols and antioxidant activity

Samples were analyzed for total phenolic compounds concentration using a modification of the Folin-Ciocalteu method (Ainsworth and Gillespie, 2007; Lee et al., 2021a). Sample extracted for flavonoids is also used for the total phenols analysis. Another microcentrifuge tube was prepared, and 135 µl of distilled water, 750 µl of Folin-Ciocalteu reagent, 50 µl of the supernatant (from sample extract), and 600 µl of Na_2_CO_3_ were added in sequence. For the blank sample, 50 µl of 80% acetone was used instead of the supernatant. Then, the mixture was vor-texed for 10 s and incubated in a water bath for 20 min at 45°C. After allowing samples to cool to room temperature, the absorbance was read at 765 nm in a microplate reader (Synergy H1, BioTek, Winooski, VT). A gallic acid standard curve was prepared, and the total phenol concentration of each sample was expressed as gallic acid equivalent (GAE) on the dry weight basis (mg GAE/g DW).

Total antioxidant activity was measured using the 2,2-diphenyl-1-picrylhydrazyl (DPPH) antioxidant assay described by Herald et al. (2012) and Alrifai et al. (2020) with slight modification. Sample extracted for flavonoids is also used for the total phenols analysis. The initial sample extraction process was similar for total phenols; however, a 1.5-ml aliquot was stored at −20°C temperature overnight. The following day, samples were centrifuged at 12,000 × *g* for 2 min; 200 µl of the DPPH (350 mM in 80% methanol) solution was combined with 25 µl of the samples or standard or 2,525 µl of the 80% methanol (for reference to calculate the amount of DPPH quenched by samples or standard). For blank, 225 µl of 80% methanol was added. The microplate was covered with parafilm and a lid and incubated in the dark at room temperature for 6 h, and the absorbance was read at 517 nm (Synergy H1, BioTek, Winooski, VT). Trolox (6-hydroxy-2,5,7,8-tetramethylchroman-2-carboxylic acid, 25–800 µM) was used as a standard for antioxidant activity, and the results were expressed as Trolox equivalent on a dry mass basis (mM TEAC/g DW).

#### 2.4.4 Phytic acid content in seeds and microgreens

The phytic acid content was analyzed using the phytic acid analysis kit from Megazyme with a slightly modified protocol (Megazyme, 2019). To measure the phytic acid loss from pea and sunflower seeds after soaking, both unsoaked and soaked seeds were freeze-dried and ground. In the case of sunflowers, the seed coat was removed before grinding. For sample extraction, freeze-dried ground samples (0.5 g) were combined with 20 vol of HCl (0.66 M) extracted overnight with shaking at 200 rpm and centrifuged at 5,500 × *g* for 20 min. The supernatant was neutralized with NaOH (0.75 M). The reagent kit sent by Megazyme was used to calculate free and total phosphorous for the enzymatic dephosphorylation. The phytic acid was calculated per the Megazyme protocol’s instruction (Megazyme, 2019). The phytic acid concentration was expressed on a dry weight basis (g/100 g DW).

### 2.5 Data analysis

All collected data were analyzed using analysis of variance in the general linear mixed model for a factorial design using R (The R Project for Statistical Computing, Vienna, Austria). Data were subjected to the model assumption before analysis. Significant means among treatments were separated using the Fisher LSD mean comparison at an alpha level of 0.05. All the treatments were also compared with the control using a series of linear contrasts.

## 3 Results

### 3.1 Effects on yield and yield components

#### 3.1.1 Peas

**Figure 1:**
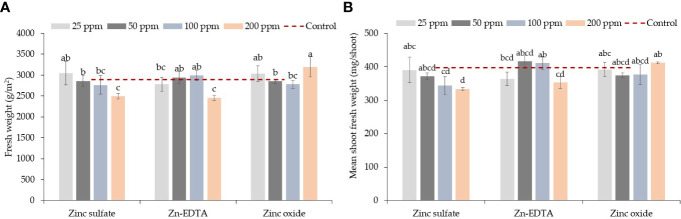
Zinc source and application rate interaction effect on pea shoot fresh weight (g/m^2^) **(A)** and mean shoot fresh weight (mg/shoot) **(B)**. Vertical bars indicate average values and the standard error (*n* = 3). Different letters indicate significant differences at *p* = 0.05 by the Fisher LSD test. The dashed line indicates the average value of the untreated control.

Zinc source and concentration levels interacted with fresh weight and mean fresh shoot weight; however, Zn source and rate had a significant effect on dry weight, and only the Zn concentration level affected dry matter content in pea microgreens (**Table 1**). ZnO at 200 ppm had higher fresh weight and mean fresh shoot weight than the ZnSO_4_ and Zn-EDTA at the same concentration (**Figures 1A, B**). A decrease in fresh weight was observed with increasing the concentrations of ZnSO_4_ and Zn-EDTA, but there was no difference between low (25 ppm) and high (200 ppm) concentrations when the seeds were soaked in the ZnO nanoparticle solutions. When compared with the control, none of the treatment combinations tested had a significantly different fresh weight or mean fresh shoot weight (**Table 1**). The pea shoot dry yield was higher for seeds soaked with ZnO than the other two sources of Zn tested, and on average, it was higher at 25 ppm of Zn compared to all the other application rates and the untreated control. Zinc source did not affect the pea shoot dry matter content, but it was higher at 25 ppm of Zn than at 50, 100, and 200 ppm of Zn.

**Table 1:** Effect of seed soaking with different Zn sources and application rates on fresh yield (g/m^2^), dry biomass (g/m^2^), dry matter content (%), and mean shoot fresh weight (mg/shoot) of pea microgreens.^1^

Pea		Fresh yield (g/m^2^)	Dry biomass (g/m^2^)	Dry matter (%)	Mean shoot fresh weight (mg/shoot)
	Zn source				
	ZnSO_4_	2,790.23	260.29 b	9.38	359.74 b
	Zn-EDTA	2,792.08	261.03 b	9.43	386.11 a
	ZnO	2,966.18	282.83 a	9.58	388.90 a
	Zn-rate (mg/l)				
	25	2,952.22	295.21 a Ϯ	10.10 a	381.94
	50	2,883.14	265.74 b	9.24 b	387.36
	100	2,845.47	259.70 b	9.16 b	377.31
	200	2,727.16	251.44 b	9.32 b	366.38
	Control	2,895.37	261.93	9.07	397.07
Source of variation	Source	ns	**	ns	*
	Rate	ns	***	*	ns
	Source × Rate	**	ns	ns	*

^1^Reported values are averages of three replications. Significance: ns = not significant, *p ≤ 0.05, **p ≤ 0.01, or ***p ≤ 0.001. Means followed by different letters within each column are significantly different at α = 0.05 via the Fisher LSD test. Ϯ indicates a significant difference compared to the control using contrast in the linear mixed model.

While comparing the Zn sources, ZnO had a higher overall fresh weight, mean shoot fresh weight, and dry biomass. Unlike ZnSO_4_ and Zn-EDTA, seed soaking at 200 ppm Zn using ZnO did not reduce fresh weight or mean fresh shoot weight (**Figures 1A, B**). Pea microgreens also had higher dry biomass when seeds were soaked in ZnO solution than ZnSO_4_ and Zn-EDTA, while Zn sources did not affect the dry matter content (**Table 1**). Except for the dry biomass of pea microgreens soaked in 25 ppm of Zn, no significant differences were observed for other yield components between all the Zn source and concentration combinations tested and the untreated control.

#### 3.1.2 Sunflower

In the case of sunflower, there was no interaction effect of the Zn source and concentration rate on fresh yield, dry yield, dry matter content, and mean fresh shoot weight (**Table 2**). The application of ZnO resulted in the highest sunflower fresh and dry biomass, followed by the application of Zn-EDTA and ZnSO_4_, respectively, while the sunflower shoot dry matter content was higher when using ZnSO_4_ compared to ZnO and Zn-EDTA. Dry matter (%) was higher when seeds were soaked with ZnSO_4_ compared to Zn-EDTA and ZnO.

**Table 2:** Effects of seed soaking with different Zn sources and rate on fresh yield, dry yield, dry matter, and fresh weight/shoot of sunflower microgreens.^1^

Sunflower		Fresh yield (g/m^2^)	Dry biomass (g/m^2^)	Dry matter (%)	Mean shoot fresh weight (mg/shoot)
	Zn source				
	ZnSO_4_	2,961.28 c	221.22 c	7.53 a	432.70
	Zn-EDTA	3,250.77 b	231.12 b	7.14 b	440.48
	ZnO	3,510.39 a	240.58 a	6.88 b	434.71
	Zn rate (mg/l)				
	25	3,320.96	237.52	7.20	429.21
	50	3,203.39	228.66	7.17	438.59
	100	3,176.96	225.29	7.14	444.28
	200	3,261.96	232.42	7.21	431.77
	Control	3,190.53	234.43	7.33	436.0
Source of variation	Source	***	***	***	ns
	Rate	ns	ns	ns	ns
	Source × Rate	ns	ns	ns	ns

^1^Reported values are averages of three replications. Significance: ns = not significant, ***p ≤ 0.001. Means followed by different letters within each column are significantly different at α = 0.05 via the Fisher LSD test.

### 3.2 Effects on microgreens mineral profile

#### 3.2.1 Peas

**Figure 2:**
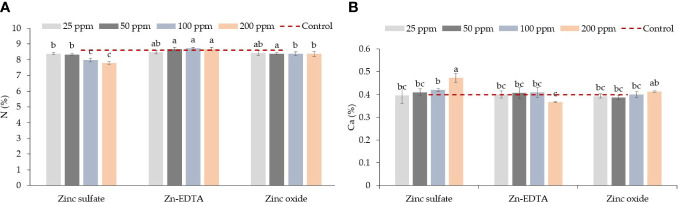
Zinc source and application rate interaction effect on total N **(A)** and Ca **(B)** content in pea microgreens (% on a dry weight basis). Vertical bars indicate average values and the standard error (*n* = 3). Different letters indicate significant differences at *p* = 0.05 by the Fisher LSD test. The dashed line indicates the average value of the untreated control.

An interaction effect between Zn source and application rate in pea shoots was seen only for N and Ca content (**Table 3**). ZnSO_4_ at 100 and 200 ppm reduced the N content compared to Zn-EDTA and ZnO at the same concentration, but on average, all Zn sources had similar N levels to the control (**Figure 2A**). Pea shoots Ca content was the highest when seeds were soaked in the ZnSO_4_ solution at 200 ppm and the lowest when seeds were soaked with Zn-EDTA at 200 ppm, while no significant differences were observed among all the other treatment combinations tested, including the untreated control (**Figure 2B**). P and K concentrations were positively affected by Zn-EDTA (**Table 3**). A Zn rate effect was observed only in the case of S, where soaking the seeds in 25 ppm of Zn resulted in higher S content in pea shoots than using 100 and 200 ppm Zn solutions, regardless of the Zn source used. Sodium concentration was not affected by the Zn source or concentration.

**Table 3:** Effects of seed soaking with different Zn sources and rate on the macromineral profile of pea microgreens.^1^

Peas		N	P	K	Ca	Mg	S	Na
	Zn source	%						
	ZnSO_4_	8.12 c	0.92 b	2.49 b	0.43 a	0.43	0.66	0.053
	Zn-EDTA	8.64 a	0.95 a	2.61 a	0.39 b	0.44	0.67	0.062
	ZnO	8.39 b	0.93 b	2.50 b	0.39 b	0.43	0.66	0.055
	Zn rate (mg/l)							
	25	8.43	0.92	2.48	0.39	0.43	0.68 a	0.051
	50	8.46	0.94	2.59	0.40	0.44	0.67 ab	0.058
	100	8.35	0.93	2.58	0.41	0.44	0.65 bc	0.062
	200	8.28	0.92	2.49	0.42	0.44	0.64 c	0.056
	Control	8.61	0.96	2.68	0.40	0.44	0.67	0.057
Source of variation	Source	***	**	*	*	ns	ns	ns
	Rate	ns	ns	ns	ns	ns	**	ns
	Source × Rate	**	ns	ns	*	ns	ns	ns

^1^Reported values are averages of three replications. Significance: ns = not significant, *p ≤ 0.05, **p ≤ 0.01, or ***p ≤ 0.001. Means followed by different letters within each column are significantly different at α = 0.05 via the Fisher LSD test.

In pea microgreens, a significant interaction effect was observed between the Zn source and concentration on the content of all the microminerals analyzed (**Table 4**). With increasing concentrations of Zn, Zn accumulation in pea shoots increased in the case of both ZnSO_4_ and ZnO, while it did not increase when Zn-EDTA was used. Compared to the control, pea seeds soaked in solutions of ZnSO_4_ at 200 and 100 ppm and ZnO applied at 200 ppm increased the pea shoot Zn content by 126%, 86.7%, and 84.7%, respectively. The increasing accumulation of Zn observed with increasing the concentration of ZnSO_4_ and ZnO nanoparticles was accompanied by a decrease in Fe content. Fe content was lower than the control at 50–200 ppm of Zn when using ZnSO_4_ and 200 ppm of Zn when using ZnO. A similar effect of the Zn accumulation was observed on Mn and Cu content, while B content increased with the Zn concentration applied when using ZnSO_4_ as a Zn source (**Table 4**).

**Table 4:** Effects of seed soaking with different Zn sources and rate on the micromineral profile of pea microgreens.^1^

Zn source × Zn rate		Mn	Fe	Cu	B	Zn
		mg/kg DW				
ZnSO_4_	25	25.00 cd	85.33 cde	13.00 abc	15.33 d	108.67 de Ϯ
	50	19.67 e Ϯ	60.00 f Ϯ	11.33 de Ϯ	17.67 abc	147.67 b Ϯ
	100	23.00 de	71.33 ef Ϯ	12.00 cd	18.33 ab	150.67 b Ϯ
	200	23.00 bc	60.00 f Ϯ	10.67 e Ϯ	19.33 a	182.33 a Ϯ
Zn-EDTA	25	28.33 abc	98.00 abc	13.33 ab	16.33 bcd	80.00 f
	50	29.00 abc	101.67 ab	14.00 a	17.67 abc	83.67 f
	100	30.67 a	105.00 a	14.00 a	16.67 bcd	86.00 f
	200	30.00 ab	108.33 a	14.00 a	15.67 cd	90.33 f
ZnO	25	28.33 abc	97.00 abc	13.67 a	16.00 cd	103.66 e Ϯ
	50	25.67 bcd	89.33 bcd	13.00 abc	15.67 cd	118.00 d Ϯ
	100	21.67 de Ϯ	75.33 def	12.00 cd Ϯ	16.67 bcd	136.00 c Ϯ
	200	21.33 de Ϯ	73.33 ef Ϯ	12.33 bcd	16.67 bcd	149.00 b Ϯ
	Control	29.00	98.00	14.00	17.00	80.67
Source of variation	Source	***	***	***	**	***
	Rate	ns	**	**	*	***
	Source × Rate	**	***	***	***	***

^1^Reported values are averages of three replications. Significance: ns = not significant, *p ≤ 0.05, **p ≤ 0.01, or ***p ≤ 0.001. Means followed by different letters within each column are significantly different at α = 0.05 via the Fisher LSD test. Ϯ indicates a significant difference compared to the control using contrast in the linear mixed model.

#### 3.2.2 Sunflowers

Zn source and concentration had no interaction effects on the macromineral profile of sunflower shoots (**Table 5**). The Zn source affected only Ca, Mg, and S concentrations, while the Zn application rate did not affect the micromineral profile of sunflower microgreens (**Table 6**). ZnSO_4_ decreased sunflower shoot Ca, Mg, and S content compared to ZnO and S content compared to Zn-EDTA but had similar concentrations of Ca and Ma compared to Zn-EDTA.

**Table 5:** Effects of seed soaking with different Zn sources and rate on the macromineral profile of sunflower microgreens.^1^

Sunflower		N	P	K	Ca	Mg	S	Na
	Zn source	%						
	ZnSO_4_	4.16	0.77	2.38	0.75 b	0.97 b	0.62 b	0.14
	Zn-EDTA	4.17	0.78	2.41	0.77 ab	0.99 ab	0.65 a	0.14
	ZnO	4.15	0.78	2.42	0.79 a	1.02 a	0.66 a	0.16
	Zn rate (mg/l)							
	25	4.15	0.77	2.35	0.78	1.0	0.67	0.14
	50	4.18	0.78	2.47	0.77	0.98	0.63	0.14
	100	4.17	0.78	2.42	0.77	0.99	0.64	0.15
	200	4.15	0.77	2.39	0.77	0.99	0.64	0.16
	Control	4.16	0.76	2.47	0.79	1.00	0.64	0.15
Source of variation	Source	ns	ns	ns	*	*	*	ns
	Rate	ns	ns	ns	ns	ns	ns	ns
	Source × Rate	ns	ns	ns	ns	ns	ns	ns

^1^Reported values are averages of three replications. Significance: ns = not significant, *p ≤ 0.05. Means followed by different letters within each column are significantly different at α = 0.05 via the Fisher LSD test.

**Figure 3:**
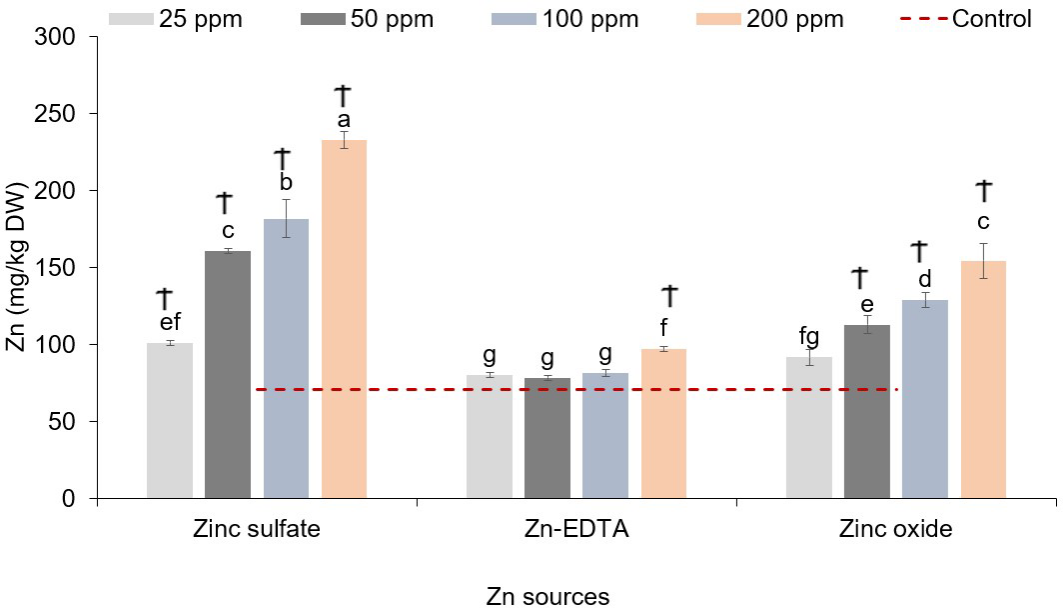
Zinc source and application rate interaction effect Zn content in sunflower microgreens (mg/kg DW). Vertical bars indicate average values and the standard error (*n* = 3). Different letters indicate significant differences at *p* = 0.05 by the Fisher LSD test. The dashed line indicates the average value of the untreated control. Ϯ indicates a significant difference compared to the control. Ϯ indicates a significant difference compared to the control using contrast in the linear mixed model.

When examining the impact of the Zn nutri-priming treatment on the sunflower shoot micromineral profile, a significant treatment effect was observed only on the Zn content. No significant interactions or main effects of Zn source and Zn concentration were observed on the sunflower microgreens’ Fe, Mn, Cu, and B contents (**Table 6**). **Figure 3** shows the interaction effect of the treatments on the Zn concentration in sunflower microgreens. Zinc accumulation increased with increasing Zn concentration in the soaking solution when ZnSO_4_ and ZnO were applied, while Zn-EDTA increased the accumulation of Zn in the sunflower shoots only when applied at 200 ppm of Zn. The highest sunflower shoot Zn accumulation was achieved by applying ZnSO_4_ at 200 ppm, followed by ZnSO_4_ at 100 ppm, and ZnO at 200 ppm, resulting in Zn contents that were 3.32, 2.58, and 2.2 times higher compared to the control, respectively.

**Table 6:** Effects of seed soaking with different Zn sources and rate on the micromineral profile of sunflower microgreens.^1^

Sunflower		Zn	Fe	Mn	Cu	B
	Zn source					
	ZnSO_4_	169.08 Ϯ	53.67	41.92	17.75	17.92
	Zn-EDTA	84.17	55.67	41.08	17.50	18.92
	ZnO	121.92 Ϯ	54.50	42.25	17.25	19.08
	Zn rate (mg/l)					
	25	90.89 Ϯ	55.88	41.67	17.78	18.33
	50	117.33 Ϯ	53.22	41.89	17.44	18.67
	100	130.56 Ϯ	53.78	41.56	17.33	19.00
	200	161.44 Ϯ	55.56	41.88	17.44	18.56
	Control	70.67	55.00	40.00	21.00	17.67
Source of variation	Source	***	ns	ns	ns	ns
	Rate	***	ns	ns	ns	ns
	Source × Rate	***	ns	ns	ns	ns

^1^ Reported values are averages of three replications. Significance: ns = not significant, ***p ≤ 0.001. Means followed by different letters within each column are significantly different at α = 0.05 via the Fisher LSD test. Ϯ indicates a significant difference compared to the control using contrast in the linear mixed model.

### 3.3 Effects on the content of phytochemicals and phytic acid

#### 3.3.1 Peas

**Figure 4:**
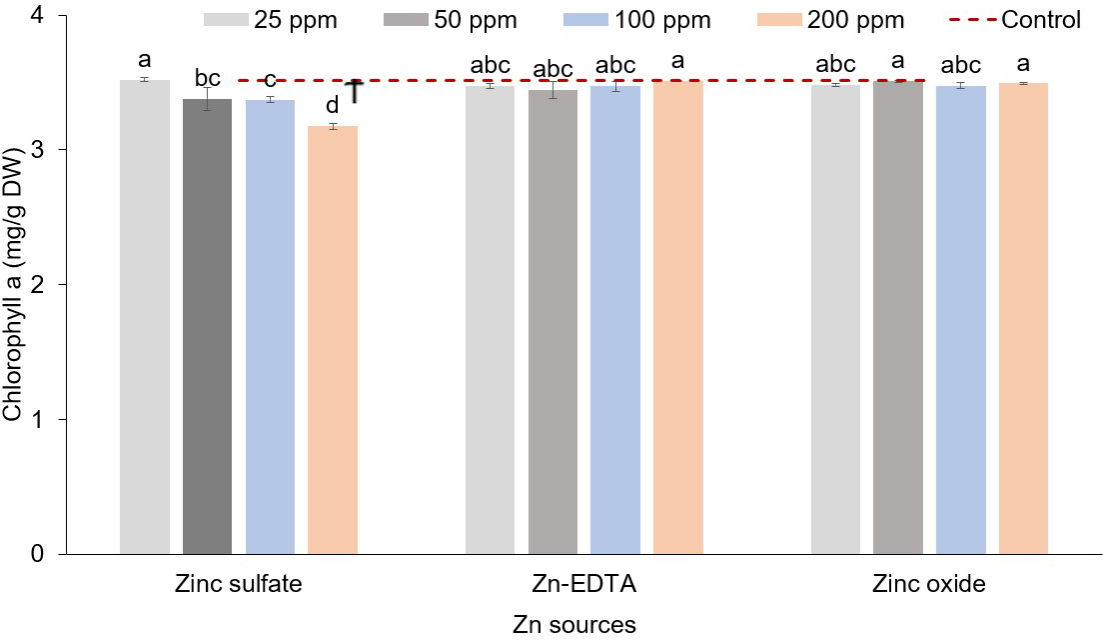
Zinc source and application rate interaction effect on chlorophyll a (mg/g DW) content in microgreens (% on a dry weight basis). Vertical bars indicate average values and the standard error (*n* = 3). Different letters indicate significant differences at *p* = 0.05 by the Fisher LSD test. The dashed line indicates the average value of the untreated control. Ϯ indicates a significant difference compared to the control using contrast in the linear mixed model.

In pea microgreens, an interaction effect between Zn source and concentration was observed on chlorophyll a, while Zn source had a significant impact on chlorophyll b and a+b (**Table 7**). Zinc source and concentration had no effect on the carotenoid concentration. Chlorophyll concentration decreased with increasing Zn rate when ZnSO_4_ was used. The application of ZnSO_4_ at the rate of 200 ppm of Zn resulted in a lower concentration of chlorophyll a (**Figure 4**). Chlorophyll b and a+b were lower when using ZnSO_4_ as a Zn fertilizer (**Table 7**). However, when all treatment combinations were compared to the control, there were no differences in chlorophyll a, chlorophyll b, chlorophyll a+b, and carotenoids. Soaking pea seeds in different sources and concentrations of Zn nutrient solution did not affect anthocyanin and flavonoid concentrations in pea microgreens (**ST1**). Both anthocyanin and flavonoid content were similar among treatments and with control.

**Table 7:** Chlorophyll a, chlorophyll b, and chlorophyll a+b, and carotenoid content in pea microgreens nutri-primed with different sources of Zn and concentration rate.^1^

Peas		Chlorophyll a	Chlorophyll b	Chlorophyll a+b	Carotenoids
	Zn source	mg/g DW			
	ZnSO_4_	3.36 b	1.71 b	4.80 b	0.44
	Zn-EDTA	3.68 a	2.23 a	5.42 a	0.38
	ZnO	3.49 a	1.95 ab	5.16 a	0.48
	Zn rate (mg/l)				
	25	3.49 a	2.09	5.30	0.41
	50	3.44 ab	1.95	5.11	0.45
	100	3.44 ab	2.05	5.21	0.42
	200	3.39 b	1.76	4.88	0.46
	Control	3.51	2.03	5.26	0.56
Source of variation	Source	***	**	**	ns
	Rate	*	ns	ns	ns
	Source × Rate	***	ns	ns	ns

^1^ Reported values are averages of three replications. Significance: ns = not significant, *p ≤ 0.05, **p ≤ 0.01, or ***p ≤ 0.001. Means followed by different letters within each column are significantly different at α = 0.05 via the Fisher LSD test.

The treatments had no interaction effect on total phenols or antioxidant activity (DPPH). The main effects of the Zn source and concentration were significant on total phenols (**ST1**), while only the Zn source influenced the pea shoot antioxidant activity (**ST1**). Zinc sulfate and Zn oxide had higher total phenols than Zn-EDTA, and these are the sources of Zn that increased Zn accumulation in pea microgreens (**Table 4**). Zinc concentrations of 25 ppm had lower antioxidant activity than 50, 100, and 200 ppm. All the treatments had similar total phenols compared to the control. Total antioxidant activity was higher with ZnO as a Zn source than with Zn-EDTA and the control (**ST1**).

**Figure 5:**
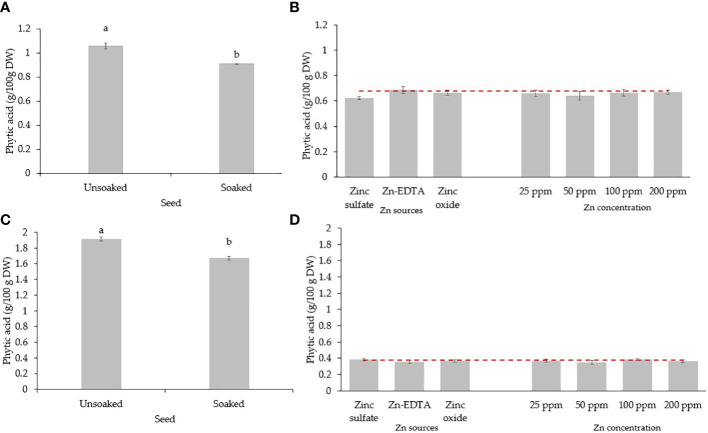
Seed soaking effect on phytic acid (g/100 g DW) content in seeds **(A)** and microgreens **(B)** of pea, and seeds **(C)** and microgreens **(D)** of sunflower. Vertical bars indicate average values and the standard error. Different letters indicate significant differences at *p* = 0.05 by the Fisher LSD test. The dashed line indicates the average value of the untreated control.

**Figure 6:**
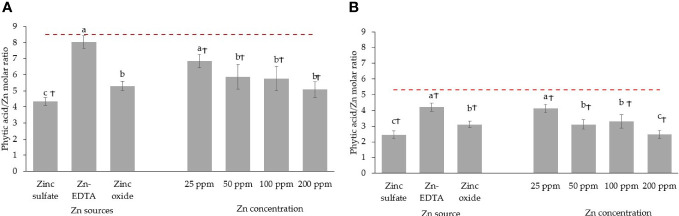
Seed soaking effect on phytic acid/Zn molar ratio in pea **(A)** and sunflower **(B)** microgreens. Vertical bars indicate average values and the standard error. Different letters indicate significant differences at *p* = 0.05 by the Fisher LSD test. The dashed line indicates the average value of the untreated control. Ϯ indicates a significant difference compared to the control using contrast in the linear mixed model.

When pea seeds were soaked in DI water overnight, the phytic acid content in the seed was reduced by 13.7% (**Figure 5A**). Soaking seeds in the Zn nutrient solution using different sources and concentrations did not affect the phytic acid content in pea microgreens (**Figure 5B**). All the treatments and the control had a similar phytic acid content, with an average phytic acid content of 0.65 g/100 g DW of pea microgreens. The phytic acid content in microgreens was lower than the phytic acid present in unsoaked (1.06 g/100 g DW) and soaked seeds (0.91 g/100 g DW) by 38.7% and 28.6%, respectively, although they were not compared statistically. The phytic acid/Zn molar ratio in the pea microgreens was lower when seeds were soaked in Zn nutrient solution from ZnSO_4_ (**Figure 6A**). Moreover, the phytic acid/Zn molar ratio in pea microgreens decreased as the rate of Zn concentration in the seed-soaking nutrient solution increased (**Figure 6A**).

#### 3.3.2 Sunflowers

In the case of sunflower shoots, the interaction and main effect of Zn concentration on chlorophyll a, chlorophyll b, chlorophyll a+b, and carotenoids were not significant. However, the source of Zn had a significant effect on the same parameters (**Table 8**). Zinc sulfate resulted in lower chlorophyll a, chlorophyll b, chlorophyll a+b, and carotenoids than Zn-EDTA and ZnO. All the treatments had similar concentrations of photosynthetic pigments compared to the control. Nutri-priming with different sources of Zn and concentration did not affect the anthocyanin and flavonoid concentration in sunflower microgreens (**ST2**).

**Table 8:** Chlorophyll a, chlorophyll b, chlorophyll a+b, and carotenoid pigments in sunflower microgreens nutri-primed with different sources of Zn and concentration rate.^1^

Sunflower		Chlorophyll a	Chlorophyll b	Chlorophyll a+b	Carotenoids
	Zn source	mg/g DW			
	ZnSO_4_	1.94 b	0.63 b	2.42 b	0.34 b
	Zn-EDTA	2.43 a	0.82 a	3.05 a	0.39 a
	ZnO	2.53 a	0.85 a	3.18 a	0.41 a
	Zn rate (mg/l)				
	25	2.09	0.70	2.63	0.35
	50	2.34	0.78	2.94	0.39
	100	2.38	0.79	2.97	0.39
	200	2.39	0.78	2.98	0.40
	Control	1.86	0.63	2.34	0.32
Source of variation	Source	*	*	*	*
	Rate	ns	ns	ns	ns
	Source × Rate	ns	ns	ns	ns

^1^ Reported values are averages of three replications. Significance: ns = not significant, *p ≤ 0.05. Means followed by different letters within each column are significantly different at α = 0.05 via the Fisher LSD test.

Zinc sources and concentration did not affect the sunflower microgreens’ total phenols and antioxidant activity (**ST2**). They also had similar total phenols and antioxidant activity to the control. Unlike pea microgreens, there was no significant interaction or main effect of seed soaking with different Zn sources and concentration levels on total phenols and antioxidant activities.

The phytic acid content in sunflower seeds decreased by 12.64% when soaked overnight in DI water (**Figure 5C**). Soaking sunflower seed with different sources and concentrations of Zn did not affect the phytic acid content in sunflower microgreens, as the phytic acid content in sunflower microgreens was similar among treatments and with the untreated control (**Figure 5D**). Just like in peas, phytic acid in sunflower microgreens (0.37 g/100 g DW) was consistently lower than in unsoaked (1.91 g/100 g DW) and soaked (1.67 g/100 g DW) sunflower seed by 80.6% and 77.8%, respectively; however, they were not compared statistically. All the seed soaking treatments reduced the phytic acid/Zn molar ratios compared to the control. A low phytic acid/Zn molar ratio was observed when ZnSO_4_ was used as a Zn source for seed soaking, followed by ZnO and Zn-EDTA (**Figure 6B**). As observed in pea microgreens, a decrease in the phytic acid/Zn molar ratio was observed with increasing the concentration of Zn in the seed-soaking solution for sunflower shoots.

## 4 Discussion

The primary objective of the present study was to assess the possibility to enrich microgreens with Zn *via* seed nutri-priming testing the effect of alternative Zn sources and application rates. Examining the effect of the treatments on two species, different responses were observed in terms of Zn accumulation, yield, impact on mineral profile, and nutritional quality. In terms of Zn enrichment, compared to the control, pea microgreens’ Zn content increased by 126% and 84.7% after soaking seeds overnight in 200 ppm of Zn solutions prepared using ZnSO_4_ and ZnO, respectively. A similar trend was observed for sunflower microgreens that recorded a 229.7% and 118.4% Zn content increase compared to the control, when seeds were soaked overnight in 200 ppm of Zn solution prepared using ZnSO_4_ and ZnO, respectively. Instead, Zn-EDTA did not determine an increase of Zn content in both microgreen species at all the application rates tested, except for a small increase at 200 ppm of Zn in the case of sunflower microgreens.

Consumption of 100 g of fresh pea microgreens biofortified with 200 ppm of ZnSO_4_ and ZnO could fulfill 21.3% and 17.6% of the RDA (recommended dietary allowance) of Zn (8 mg for adults (IMFN, 2001), respectively. In the case of sunflower microgreens, the consumption of 100 g of fresh shoots biofortified with 200 ppm of ZnSO_4_ and ZnO could fulfill 21.5% and 13.6% of the Zn RDA for an adult (IMFN, 2001). According to the FDA Regulatory Requirements for Nutrient Content Claim (Boon et al., 2010; FDA, 2022), food that fulfills (per serving) 20% or more of the RDA of a certain nutrient is considered a “high source” of that nutrient, and food that fulfills 10%–19% of the RDA is considered a “good source”. The increase of Zn content obtained with the application of ZnSO_4_ and ZnO *via* seed nutri-priming suggests that both fertilizer sources applied at 200 ppm may allow the production of Zn-enriched pea and sunflower microgreens that could be a “good” or “high” source of Zn. Nevertheless, the selection of the optimal source and application rate of Zn for pea microgreen biofortification should take into consideration not only the increase of Zn content but also the effect on crop yield and quality, including effects on other minerals.

Seed soaking in solutions prepared with different sources and concentrations of Zn had significant effects on the yield components of pea microgreens. Fresh weight and mean fresh shoot weights were lower when seeds were soaked at 200 ppm of ZnSO_4_, which could be due to Zn toxicity. The mean fresh shoot weight and fresh weight both followed a similar pattern. Dry biomass and dry matter content were reduced when Zn concentrations applied exceeded 25 ppm. Visual symptoms of Zn toxicity manifested with a pale-green color of the leaves only in the case of ZnSO_4_ applied at 200 ppm but not with Zn-EDTA, although Zn-EDTA applied at 200 ppm caused a slight decrease in fresh weight. No Zn toxicity symptoms were observed also when using ZnO nanoparticles as a Zn source. The pale-green color or light yellowing symptom seen in the case of seed soaked into a 200 ppm of ZnSO_4_ solution is consistent with the low levels of chlorophyll a (**Figure 4**), total N (**Figure 2A**), and Fe (**Table 4**) observed in the same pea microgreens. Zn toxicity can cause chlorosis symptoms, as the excess of Zn impairs the photosynthetic function and reduces chlorophyll and N levels in leaves (Broadley et al., 2007; Balafrej et al., 2020). The effect of the excess Zn on yield components and the reduction of chlorophyll content can also be related to the reduction of Fe content, as increasing Zn concentration has been found to reduce Fe uptake in many cases (Di Gioia et al., 2019; Sahin, 2021), and Fe is an important component of chlorophyll pigments. Thus, we can infer that the application of excess Zn may have caused decreased rates of Fe and N uptake and a reduction in chlorophyll content, impairing the photosynthetic activity, which, in turn, reduced plant growth. Enrichment of seeds with Zn through nutri-priming generally increases the fresh yield and dry biomass by increasing germination, seed vigor, and establishment, and improved plant–water relations (Prom-u-thai et al., 2012; Montanha et al., 2020). However, above a certain threshold, which may be crop-specific, Zn enrichment can negatively affect crop yield (Bączek-Kwinta et al., 2020). Bączek-Kwinta et al. (2020) used random amplification of polymorphic DNA (RAPD) markers to evaluate the effect of Zn on sprouts of different species and observed higher DNA variation in pea sprouts when seeds were soaked overnight in 30 ppm of Zn solution compared to the seeds soaked in Zn-free solution. The higher DNA variation observed using RAPD markers in sprouts soaked at 30 ppm of Zn was related to Zn toxicity. Information on the effect of seed soaking with different Zn sources on the production parameters of microgreens is limited. However, Singh et al. (2016) found higher seed germination, seed vigor, and plumule length when they soaked tomato seed in ZnO nanoparticle solutions as compared to ZnSO_4_, although they used ZnO nanoparticles extracted from the Russian olive flower (*Elaeagnus angustifolia* L.), unlike the synthetic form of nanoparticles we used in this study. Besides the variations of total N and Fe in response to the Zn source and application rate, the application of Zn enhanced Ca accumulation only in the case of ZnSO_4_ and slightly in the case of ZnO (**Figure 2B**). Such an effect would be seen only if the application of Zn increased Zn accumulation, as we did not see an increase in Ca content in the case of Zn-EDTA (**Table 4**). Higher Ca accumulation along with Zn was also reported by Di Gioia et al. (2019) in Brassica microgreen species. Likewise, Zou et al. (2014) found higher Ca in soybean sprouts when they soaked and grew soybean sprouts at different Zn concentrations (20–100 ppm) compared to the untreated control. The synergistic effect of Zn on Ca accumulation was also reported by Abbas et al. (2017). However, a negative relationship between Zn and Ca has also been discussed as they compete for absorption sites in root regions (Rugeles-Reyes et al., 2019), and this relationship has been found to change based on the species (Lingyun et al., 2016; D’Imperio et al., 2022). Soaking seeds in the Zn-EDTA nutrient solution resulted in higher P and K compared to soaking seeds in ZnSO_4_ and ZnO solutions. Such an effect can also be linked to the effect of Zn on P uptake as Zn could reduce P accumulation in plants (Barrameda-Medina et al., 2017). Zn-EDTA did not increase Zn content at any concentration applied in this study, unlike ZnSO_4_ and ZnO (**Table 4**). Such an effect could be explained by a lower seed permeability and absorption of Zn-EDTA compared to the other two sources of Zn.

The observed antagonistic effect of Zn accumulation on other divalent cations like Fe, Mn, and Cu is generally expected as they share the same groups of transporters, including the ZIP (ZRT IRT- like protein), NRAMP (Natural Resistance-Associated Macrophage Protein), and yellow stripe-like (YSL) families for mineral absorption at the root level and translocation within plants (Eide et al., 1996; Kaur and Garg, 2021). Di Gioia et al. (2019), Preciado-Rangel et al. (2021), and Sahin (2021) have also reported a decrease in Fe content with increasing Zn accumulation in Brassica microgreens and lettuce. Similarly, the antagonistic effect of Zn accumulation on Mn and Cu content was reported by Preciado-Rangel et al. (2021) and Sahin (2021) in lettuce. As Fe and Zn are the major deficient minerals for human health (Zalewska and Nogalska, 2014), the decrease of Fe content observed with increasing Zn accumulation is not ideal for human nutrition and the selection of the optimal Zn treatment should consider the trade-off between Zn accumulation and Fe decrease. Future research should consider the simultaneous biofortification with Fe and Zn considering that both micronutrients are deficient in large portions of the global population.

In the case of sunflower microgreens, the concentration of Zn applied with any of the Zn sources did not affect any of the yield components examined, and considering the relatively high levels of Zn accumulated in the shoots, this study corroborates previous findings, suggesting that sunflower is a hyperaccumulator of Zn (Adesodun et al., 2010). In fact, sunflower has been studied for the phytoremediation of Zn-contaminated soil (Zalewska and Nogalska, 2014; Soares et al., 2021). The higher dry matter (%) content observed in seeds primed with ZnSO_4_ could be explained by the potential stress caused by the higher Zn levels accumulated in microgreens when seeds were soaked in ZnSO_4_ solution (**Figure 3**). However, in the case of sunflower microgreens, there were no visible symptoms of Zn phytotoxicity or stress with any of the Zn sources and concentration levels tested.

Unlike pea microgreens, Zn application through seed soaking and its accumulation in plants had no or very limited effect on the macro- and micromineral profile of sunflower shoots. Such results are consistent with the capacity of sunflowers to absorb relatively large quantities of Zn without causing phytotoxicity and any major plant physiological disruption leading to effects on the uptake and accumulation of other minerals. In general, antagonistic effects between Zn and other divalent cations are expected; however, such effects differ based on the ability of the crop to tolerate Zn excess (Rout and Das, 2009). The negative impact of Zn accumulation on other microminerals may not be observed in a Zn hyperaccumulator such as sunflower, while it has been observed on other crops like peas in this study. The lack of effects of Zn accumulation on the concentration of other microminerals has been reported by Rugeles-Reyes et al. (2019) in arugula when they applied different concentrations of Zn through a foliar spray.

Seeds soaked in solutions prepared with different sources and concentrations of Zn resulted in pea microgreens with similar chlorophyll a, chlorophyll b, and chlorophyll a+b content compared to the seeds soaked in DI water. These results are in contrast with the findings of Lingyun et al. (2016) on pea sprouts, as they found higher chlorophyll a, chlorophyll b, and chlorophyll a+b when seeds were soaked overnight in a nutrient solution of Zn (10–60 ppm). However, in the present study, a decrease of these pigments was observed only with the application of ZnSO_4_ at 100 and 200 ppm of Zn. Other studies on green beans (Bautista-Diaz et al., 2021) and arugula (Rugeles-Reyes et al., 2019) did not show an increment in chlorophyll pigments and carotenoids with the accumulation of Zn in plant tissues. An increase in chlorophyll a and chlorophyll b concentration with the use of ZnO nanoparticles compared to ZnSO_4_ was also reported by Bautista-Diaz et al. (2021) as observed in the present study; nevertheless, Bautista-Diaz et al. (2021) applied Zn through foliar spray. When pea microgreens were soaked in solutions containing higher concentrations of Zn (50–200 ppm), total phenolic compounds increased. The increase in phenolic compounds was observed only when there was an increased accumulation of Zn. In fact, unlike Zn-EDTA, only ZnSO_4_ and ZnO increased Zn accumulation with the increase in Zn application rates, and these are the sources of Zn that determined an increase in phenolic compounds and antioxidant activity (**ST1**). The increased content of total phenolics and antioxidant activity could be associated with a defense mechanism of the plant in response to the stress caused by the accumulation of Zn in plant tissues at toxic levels (Michalak, 2006; Chen et al., 2019). Alternatively, the higher availability of Zn favored the biosynthesis of phenolic compounds considering that Zn is a cofactor required for various metabolic pathways that lead to the biosynthesis of phenolic compounds (Castillo-González et al., 2018). These results were consistent with the findings of Lingyun et al. (2016); Barrameda-Medina et al. (2017), and Preciado-Rangel et al. (2021) who, while working on pea sprouts, cabbage, and lettuce, respectively, observed an increase in total phenolic compounds and antioxidant activity when the amount of Zn in plant increased.

In the case of sunflower, only the Zn sources affected chlorophyll a, chlorophyll b, chlorophyll a+b, and carotenoids, as those parameters were higher when ZnO and Zn-EDTA were used as a Zn source. Bautista-Diaz et al. (2021) also reported higher levels of chlorophyll a and chlorophyll b in the leaves of beans when they used ZnO as a Zn source compared to ZnSO_4_, although they applied Zn through foliar application. No effects were observed on the total phenolics content of sunflower microgreens. It is possible that, in sunflower, the accumulation of Zn may not have determined a stress and did not influence the synthesis of phenolic compounds and the antioxidant activity. Such results may be explained by the ability of sunflower and other hyperaccumulator plants to accumulate heavy metals such as Zn (Chae et al., 2014; Dhiman et al., 2017). Hyperaccumulator plants store Zn in plant tissue through different mechanisms, like compartmentalization of the vacuoles (Clemens, 2017).

An important aspect to consider in the selection of target crops for mineral biofortification is the presence of anti-nutritional factors that may limit the bioavailability of micronutrients. In many cereal and leguminous crops used for biofortification purposes, the presence of phytate in seeds can substantially limit the bioavailability of micronutrients; instead, often, it has been claimed that sprouts and microgreens are particularly suitable for micromineral enrichment because of the relatively low content of phytate (Di Gioia et al., 2019; Galieni et al., 2020). While limited information is available on microgreens, the present study demonstrates that during the process of seed soaking and germination, the content of phytate is substantially decreased. Soaking seeds mainly reduces the phytic acid concentration in two ways: (i) through leaching and (ii) through biosynthesis and activation of the phytase enzymes (Egli et al., 2002; Feizollahi et al., 2021). Phytic acid is mostly soluble in water; thus, it is easy to reduce its content in seeds through leaching (Gupta et al., 2015); however, leaching is more effective in monocot than in dicot seeds as phytate is mostly stored in germplasm, the aleurone layer in monocot seeds, and germplasm and cotyledon in dicot (Raboy, 2009; Feizollahi et al., 2021). The biosynthesis of the phytase, which could reduce phytic acid through reduction, is also possible; however, a 12-h soaking period may be too short to see the effects of the phytase activity. Instead, the biosynthesis and activity of the phytase usually occur more during the actual seed germination (Gibson and Ullah, 1988). In the present study, a 13.7% reduction in phytic acid was observed in pea seeds when seeds were soaked in DI water for 12 h, which is a lower reduction percentage compared to the reduction reported (30%) by Egli et al. (2002) and greater than the value reported (3.94%) by EL-Suhaibani et al. (2020); however, they soaked seeds for 16 and 6 h, respectively. The amount of phytic acid reduction through soaking depends on several factors, including temperature, duration, and pH of the soaking medium (Feizollahi et al., 2021). A similar reduction of phytic acid (by 12.64%) was observed in sunflower seeds after soaking in DI water for 12 h. Egli et al. (2002) have also reported a 9% decrease in phytic acid in sunflower seeds after soaking seeds for 16 h under dark conditions. The lower level of phytic acid observed in this study in pea microgreens on a dry mass basis compared to the seeds before and after soaking was expected, considering that during the germination process, generally there is a reduction of the phytic acid due to the phytase biosynthesis and potentially to a dilution effect with the growth of microgreen shoots (Di Gioia et al., 2017; Mehanni et al., 2017).

Besides the reduction of phytate during the soaking and germination process, it is possible to hypothesize that the short growing cycle of microgreens limits the accumulation of antinutrients like phytate compared to their mature counterpart (Di Gioia et al., 2017; Teng et al., 2021). Although there was no difference in phytic acid concentration in pea and sunflower microgreens, a significant treatment effect was observed on the phytic acid/Zn molar ratio, which decreased with the accumulation of Zn in plant tissue. In both species, the phytic acid/Zn molar ratio decreased with the use of ZnSO_4_ and ZnO as a Zn source and with the increase in the concentration of Zn applied. The decrease of phytic acid/Zn molar ratio was due to the increased accumulation of Zn associated with these treatments rather than to the variation of phytic acid. A lower value of the phytic acid/Zn molar ratio suggests a higher Zn bioavailability and bioaccessibility in biofortified pea and sunflower microgreens (Morris and Ellis, 1989). However, further research is needed to assess the actual *in vitro* and *in vivo* bioaccessibility and bioavailability of Zn in biofortified pea and sunflower microgreens. Moreover, limited information is available on the potential effects of ZnO nanoparticles and Zn-EDTA residues on human health and a dedicated research effort is needed to assess such effects before implementing Zn biofortification treatment on microgreens at the commercial level.

## 5 Conclusions

The results of the present study reveal that seed nutrient priming in peas and sunflower microgreens is an effective Zn biofortification method. Zinc sulfate, followed by ZnO at 200 ppm, was the best source of Zn, and sunflower microgreens accumulated more Zn (229.8%) than peas (126.1%). An antagonistic effect on the accumulation of other micronutrients (Fe, Mn, and Cu) was seen only in pea microgreens. Furthermore, seed nutrient primimg in the case of peas and sunflowers reduced the amount of phytic acid, an antinutrient that limits the bioaccessibility of zinc, thereby improving Zn bioavailability. However, excessive Zn application can negatively affect the content of other minerals, such as Fe. Zinc nutrient priming increased total phenolics and antioxidant activity in peas while having no effects on sunflower microgreens. Further research is needed to evaluate the efficacy of alternative agronomic biofortification approaches and to identify other zinc hyper-accumulator species that can be enriched with Zn while minimizing negative effects on yield and the content of other essential nutrients.

## Data availability statement

The original contributions presented in the study are included in the article/**Supplementary Material**. Further inquiries can be directed to the corresponding author/s.

## Author contributions

Conceptualization, PP and FD; Methodology, PP and FD; Software, PP; Validation, FD; Formal Analysis, PP; Investigation, PP; Resources, FD; Data curation, PP; Writing—Original Draft Preparation, PP; Writing—Review and Editing, FD, EC, and JL; Supervision, FD; Project Administration, FD; Funding Acquisition, FD. All authors contributed to the article and approved the submitted version.
