# Supplementary material for: Zinc biofortification through seed nutri-priming using alternative zinc sources and concentration levels in pea and sunflower microgreens
Source: Front Plant Sci. 2023 Apr 17;14:1177844. doi: 10.3389/fpls.2023.1177844 (PMC10150129; doi:10.3389/fpls.2023.1177844)
Supplement: Supplementary file 1 [file Table_1.docx]

Supplementary Material

Zinc Biofortification Through Nutri-Priming Using Alternative Zinc Sources and Concentration Levels in Grey Sugar Pea and Black Oil Sunflower Microgreens

**Pradip Poudel, Francesco Di Gioia *, Joshua D. Lambert, and Erin L. Connolly**

*** Correspondence:** Francesco Di Gioia: [fxd92@psu.edu](mailto:fxd92@psu.edu)

# Supplementary Data

**Supplementary Table 1 (ST1): Anthocyanin, flavonoids, total phenols, and total antioxidants content in pea microgreens nutri-primed with different sources of Zn and concentration rate.^1^**

| Peas |  | Anthocyanin (Abs530/g DW) | Flavonoids  (mg CE/g DW) | Total phenols (GA mg/g DW.) | Total antioxidant  (Trolox (µM)/g DW.) |
| --- | --- | --- | --- | --- | --- |
|  | Zn source |  | |  | |
|  | ZnSO_4_ | 20.90 | 7.42 | 11.95 a | 44.17 ab |
|  | Zn-EDTA | 25.14 | 7.30 | 10.64 b | 41.02 b |
|  | ZnO | 24.26 | 8.40 | 11.72 a | 48.64 a Ϯ |
|  | Zn rate (mg/L) |  |  |  |  |
|  | 25 | 24.69 | 7.85 | 10.45 b | 41.83 |
|  | 50 | 27.59 | 7.66 | 11.66 a | 43.05 |
|  | 100 | 21.19 | 7.78 | 11.65 a | 44.98 |
|  | 200 | 20.27 | 7.55 | 11.94 a | 48.58 |
|  | Control | 20.58 | 7.63 | 10.45 | 34.35 |
| Source of variation | Source | ns | ns | * | ** |
|  | Rate | ns | ns | ** | ns |
|  | Source×Rate | ns | ns | ns | ns |

^1^ Reported values are averages of three replications. Significance: ns=not significant, * P ≤0.05 and ** P≤0.01, respectively. Means followed by different letters within each column are significantly different at α = 0.05 via the Fisher L.S.D. test. Ϯ indicates a significant difference compared to the control using contrast in linear mixed model.

Supplementary Table 2 (ST2): Anthocyanin, flavonoids, total phenols, and total antioxidants content in sunflower microgreens nutri-primed with different sources of Zn and concentration rate.^1^

| Sunflowers |  | Anthocyanin (Abs530/g DW) | Flavonoids  (mg CE/g DW) | Total phenols (GA mg/g DW) | Total antioxidant  (Trolox (µM)/g DW) |
| --- | --- | --- | --- | --- | --- |
|  | Zn source |  | |  | |
|  | ZnSO_4_ | 14.13 | 6.76 | 8.89 | 76.80 |
|  | Zn-EDTA | 12.65 | 5.94 | 7.67 | 67.91 |
|  | ZnO | 11.10 | 7.18 | 8.70 | 78.8 |
|  | Zn rate (mg/L) |  |  |  |  |
|  | 25 | 12.52 | 6.77 | 8.72 | 77.18 |
|  | 50 | 12.43 | 6.89 | 8.00 | 72.54 |
|  | 100 | 13.89 | 6.42 | 8.61 | 74.67 |
|  | 200 | 11.67 | 6.42 | 8.35 | 72.81 |
|  | Control | 11.10 | 9.36 | 8.56 | 78.10 |
| Source of variation | Source | ns | ns | ns | ns |
|  | Rate | ns | ns | ns | ns |
|  | Source×Rate | ns | ns | ns | ns |

^1^ Reported values are averages of three replications. Significance: ns=not significant. Means followed by different letters within each column are significantly different at α = 0.05 via the Fisher L.S.D. test.
